# Pridopidine subtly ameliorates motor skills in a mouse model for vanishing white matter

**DOI:** 10.26508/lsa.202302199

**Published:** 2024-01-03

**Authors:** Ellen Oudejans, Diede Witkamp, Gino V Hu-A-Ng, Leoni Hoogterp, Gemma van Rooijen-van Leeuwen, Iris Kruijff, Pleun Schonewille, Zeinab Lalaoui El Mouttalibi, Imke Bartelink, Marjo S van der Knaap, Truus EM Abbink

**Affiliations:** 1 Child Neurology, Emma Children’s Hospital, Amsterdam Leukodystrophy Center, Amsterdam University Medical Centers , Vrije Universiteit and Amsterdam Neuroscience, Amsterdam, Netherlands; 2 Department of Integrative Neurophysiology, Center for Neurogenomics and Cognitive Research, VU University, Amsterdam, Netherlands; 3 Department of Pharmacy and Clinical Pharmacology, Amsterdam UMC, Location VUmc, Amsterdam, Netherlands

## Abstract

The leukodystrophy vanishing white matter (VWM) is characterized by chronic and episodic acute neurological deterioration. Curative treatment is presently unavailable. Pathogenic variants in the genes encoding eukaryotic initiation factor 2B (eIF2B) cause VWM and deregulate the integrated stress response (ISR). Previous studies in VWM mouse models showed that several ISR-targeting compounds ameliorate clinical and neuropathological disease hallmarks. It is unclear which ISR components are suitable therapeutic targets. In this study, effects of 4-phenylbutyric acid, tauroursodeoxycholic acid, or pridopidine (PDPD), with ISR targets upstream or downstream of eIF2B, were assessed in VWM mice. In addition, it was found that the composite ataxia score represented motor decline of VWM mice more accurately than the previously used neuroscore. 4-phenylbutyric acid and tauroursodeoxycholic acid did not improve VWM disease hallmarks, whereas PDPD had subtle beneficial effects on motor skills. PDPD alone does not suffice as treatment in VWM mice but may be considered for combination therapy. Also, treatments aimed at ISR components upstream of eIF2B do not improve chronic neurological deterioration; effects on acute episodic decline remain to be investigated.

## Abstract (precis)

Assessment of the therapeutic efficacy of 4-PBA, TUDCA, and pridopidine in a vanishing white matter mouse model shows that only pridopidine subtly ameliorates motor skills.

## Introduction

Vanishing white matter (VWM) is one of the more prevalent leukodystrophies (Hamilton et al, 2018). The disease course consists of chronic neurological decline in combination with stress-provoked episodes of rapid severe deterioration, which are in part reversible. VWM leads to premature death and no curative treatment is presently available. Brain pathology is characterized by white matter rarefaction, deficient myelin, lack of adequate astrogliosis, mislocalized Bergmann glia, and immature astrocytes and oligodendrocytes in the white matter (Dietrich et al, 2005; Bugiani et al, 2011; Dooves et al, 2018). Astrocyte dysfunction is central in VWM pathogenesis (Dooves et al, 2016). The disease is caused by bi-allelic pathogenic variants in the genes encoding the five subunits (α-ε) of the eukaryotic initiation factor 2B (eIF2B) (van der Knaap et al, 2002). eIF2B is the guanine nucleotide exchange factor for the trimeric eIF2 and essential for mRNA translation and protein synthesis regulation (Konieczny & Safer, 1983; Wortham et al, 2014). In addition, eIF2B directs the integrated stress response (ISR) triggered by various types of cellular stress that phosphorylate the α-subunit of eIF2 (Proud, 2001). Phosphorylated eIF2 inhibits eIF2B, thereby reducing bulk protein synthesis rates and stimulating the production of transcription factors ATF4 and CHOP that modulate the expression of stress-ameliorating genes (Pakos-Zebrucka et al, 2016). In the case of ER stress or ER dysfunction, the ISR is activated as part of the unfolded protein response, a particular quality control system of ER function (Wang & Kaufman, 2012).

VWM pathogenic variants reduce eIF2B activity and deregulate the ISR. Transcription factors ATF4 and CHOP and their transcriptomes are up-regulated, whereas levels of phosphorylated eIF2α are low relative to control WT mice (Abbink et al, 2019). In brains from VWM patients and VWM mouse models, the ISR is progressively deregulated in astrocytes (Abbink et al, 2019; Wong et al, 2019; Terumitsu-Tsujita et al, 2020). In several preclinical studies, eIF2B has emerged as a viable drug target to ameliorate VWM (Abbink et al, 2019; Wong et al, 2019). Dampening the deregulated expression of the ATF4- and CHOP-regulated transcriptomes improves the clinical phenotype and neuropathology and decreases ISR markers (Abbink et al, 2019; Wong et al, 2019; Witkamp et al, 2022).

In the present study, we assess the response to three compounds in the clinically representative *2b4*^*he*^*2b5*^*ho*^ VWM mouse model: 4-phenylbutyric acid (4-PBA), tauroursodeoxycholic acid (TUDCA), and pridopidine (PDPD). Therapy is urgently needed in VWM. 4-PBA and TUDCA are FDA-approved, PDPD received an FDA fast-track designation and all three compounds have improving effects in other neurological disorders (Gardian et al, 2005; Ryu et al, 2005; Ricobaraza et al, 2009; Squitieri et al, 2015; Elia et al, 2016; Waters et al, 2018). 4-PBA and TUDCA are chemical chaperones with similar modes of action. They assist in protein folding in the ER and counteract the accumulation of misfolded proteins (Hetz et al, 2013). As such, the compounds are expected to increase eIF2B activity upstream in the ISR via reducing eIF2α phosphorylation (Galán et al, 2014; Yoon et al, 2016). PDPD is a sigma-1 receptor (S1R) agonist that improves motor function in a mouse model for Huntington disease (Squitieri et al, 2015). S1R is localized at the interface between the ER and mitochondria (Hayashi, 2019). The S1R has recently been discovered as a promising target for VWM treatment by correcting mitochondrial impairments in VWM fibroblasts and astrocytes (Raini et al, 2017; Atzmon et al, 2018). Moreover, S1R expression is up-regulated by ATF4 and prevents ISR-induced apoptosis without affecting eIF2α phosphorylation levels, suggesting that PDPD targets the ISR downstream of eIF2B (Mitsuda et al, 2011). We investigated how each compound affected clinical signs, neuropathological hallmarks, and the ISR in VWM mice. Control WT mice were included to identify VWM disease markers.

A sensitive scoring method of progressive clinical signs is important to demonstrate treatment effects and to determine the optimal age for measuring motor skills in balance beam and CatWalk tests. The phenotypic parameters of the neuroscore that we thus far used (Hatzipetros et al, 2015; Abbink et al, 2019; Witkamp et al, 2022) follow a sequential order (Hatzipetros et al, 2015) that does not completely match the disease course in VWM mice: we observed that the first parameter appears later in the disease course than the last parameter (unpublished observation). Therefore, an additional scoring method for neurological decline was included in the present study to determine which method assesses VWM disease course most accurately. This previously described method (Guyenet et al, 2010) uses a composite ataxia score (CAS) of four different measures taken in parallel and sensitively quantified the severity of cerebellar ataxia in a mouse model of spinocerebellar ataxia type 7, making it a promising method to assess this clinical hallmark in VWM mice.

## Results

### PDPD partly ameliorates clinical signs of VWM mice, whereas 4-PBA and TUDCA do not

**Figure 1.**
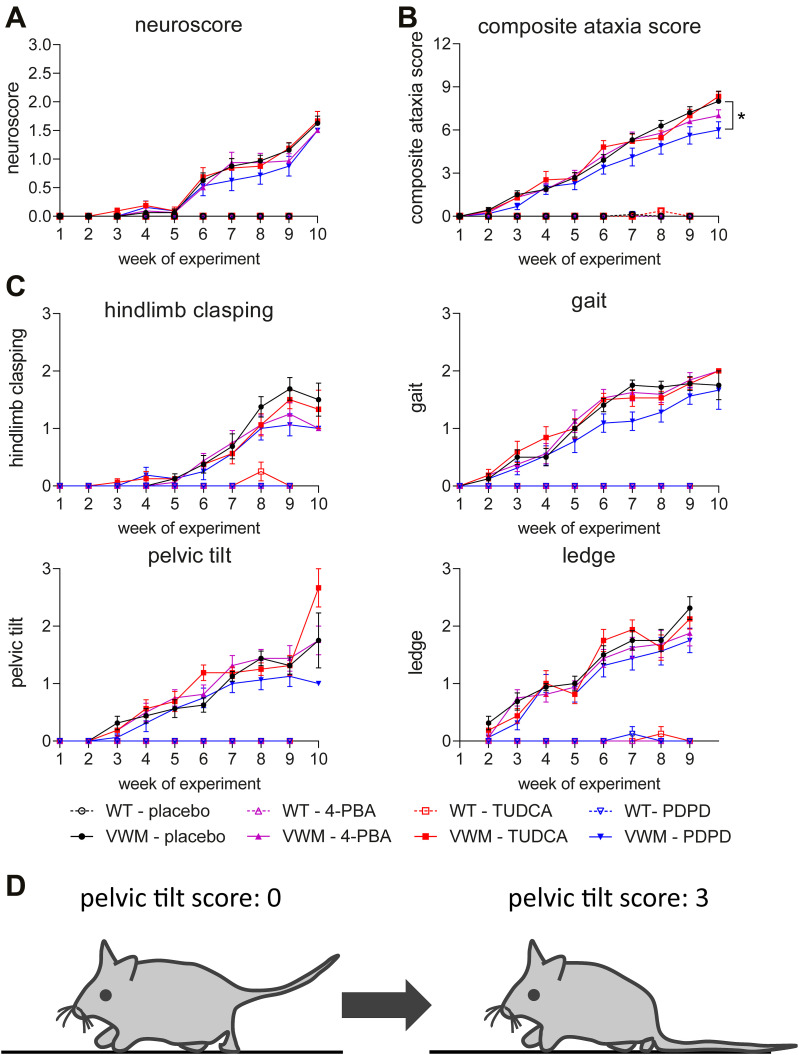
PDPD slightly ameliorates ataxia as measured by the refined CAS phenotypic scoring method. WT (open symbols) and *2b4*^*he*^*2b5*^*ho*^ (VWM, closed symbols) mice were injected daily with placebo (black circles), 500 mg/kg TUDCA (red squares), 120 mg/kg 4-PBA (magenta triangles) or 6 mg/kg PDPD (blue inverted triangles) from an age of 7–8 wk onwards for 9–10 wk (n = 8 WT mice and n = 16 VWM mice per treatment). **(A, B, C)** Graphs show neurological decline measured weekly with the neuroscore protocol (A), total CAS (B), or individual components of the CAS (C). During the first week of the experiment, ledge performance was habituated and therefore not scored. Each component is scored from 0 to 3 and the sum forms the total CAS. Graphs show mean scores ± SEM (A, B, C). **(D)** Illustrative figure of the pelvic tilt, a mouse without phenotype (left) and one with the highest score (right), the scoring protocols are provided in Supplemental Datas 1 and 2. Neuroscore and CAS differed significantly in placebo-treated WT versus placebo-treated VWM mice (*P* < 0.05; not indicated). Statistical analyses examining WT–VWM differences in placebo-treated mice in neurological decline per week were performed with paired *t* tests. **(A, B)** Statistical analyses investigating compounds-related differences in neurological decline over time were performed per genotype with a repeated measures one-way ANOVA followed by a post hoc Dunn’s correction (A, B), *P < 0.05.

**Figure 2.**
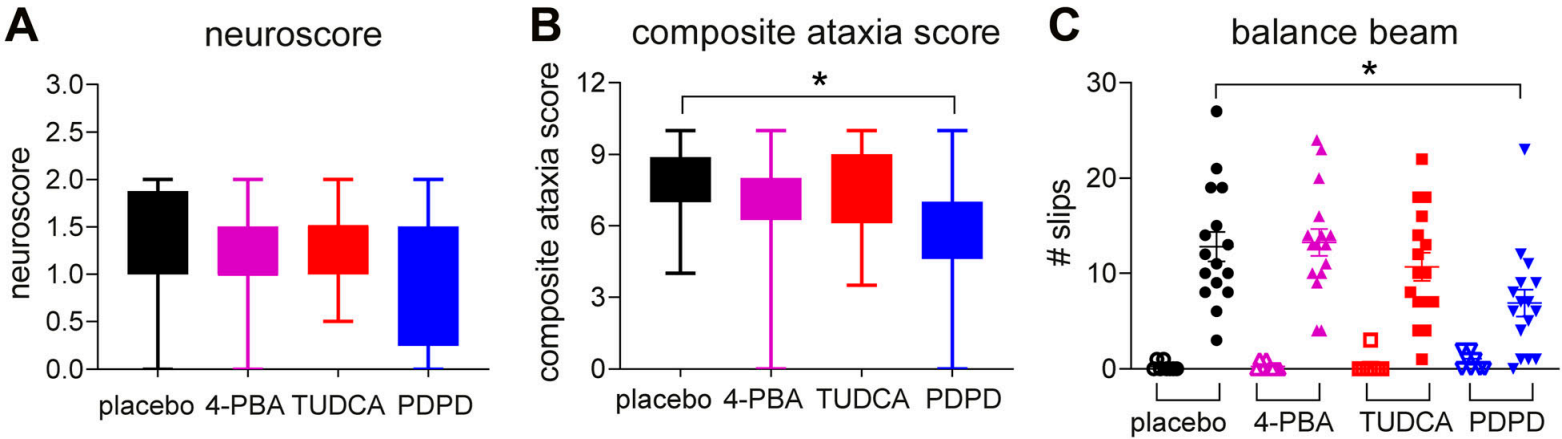
Subtle disease amelioration by PDPD detected by CAS and confirmed by balance beam performance. WT and *2b4*^*he*^*2b5*^*ho*^ (VWM) mice were injected daily with placebo (black [circles]), 500 mg/kg TUDCA (red [squares]), 120 mg/kg 4-PBA (magenta [triangles]) or 6 mg/kg PDPD (blue [inverted triangles]) from an age of 7–8 wk onwards for 9–10 wk (n = 8 WT mice and n = 16 VWM mice per treatment). **(A, B, C)** Neurological decline on the day of termination with the indicated protocols was plotted (A, B) to allow comparison with the results obtained with balance beam performance measured by number of slips (C). **(A, B, C)** Graphs show box plots with min to max of VWM mice (A, B) or mean scores ± SEM with individual data points of WT (open symbols) and VWM (closed symbols) mice (C). Neuroscore, CAS, and balance beam differed significantly in placebo-treated WT versus placebo-treated VWM mice (*P* < 0.05; not indicated). **(A, B, C)** Statistical analyses examining WT–VWM differences in placebo-treated mice in neurological decline (A, B) or balance beam performance (C) were performed with Mann–Whitney tests. **(A, B)** Statistical analyses examining compound-related differences in neurological decline in VWM mice were analyzed with a one-way ANOVA (A) or Kruskal–Wallis test followed by Dunn’s correction (B). **(C)** Statistical analysis for treatment effects on balance beam performance was performed with a Kruskal–Wallis test for WT mice and a one-way ANOVA followed by Dunnett’s multiple comparison test for VWM mice (C), *P < 0.05.

WT and VWM mice were injected daily with TUDCA, 4-PBA, PDPD, or placebo from an age of 7–8 wk onwards. At this age, VWM mice did not express clinical neurological features, and white matter damage was expected to be subtle with evident ISR deregulation in astrocytes (Dooves et al, 2016, 2018; Abbink et al, 2019). Neurological decline was assessed weekly with the neuroscore and CAS (Fig 1A and B) (Supplemental Datas 1 and 2). Both protocols showed that neurological decline significantly increased in placebo-treated VWM mice compared with placebo-treated WT mice during the experiment (NS: increase >1 out of 3, *P* = 0.0163; CAS: increase >7 out of 12, *P* = 0.00027; Supplemental Data 3; Fig 1A and B). PDPD significantly ameliorated ataxia assessed with the CAS over the course of the experiment (*P* = 0.017; Fig 1B) and during the last week of the experiment (*P* = 0.0172; Fig 2B). This amelioration was not observed with the neuroscore (Figs 1A and 2A). 4-PBA and TUDCA did not have an ameliorating effect on neurological decline in VWM mice as assessed with either protocol (Fig 1A and B). Ataxia was not observed in WT mice for any treatment with either protocol (Fig 1A–C).

Supplemental Data 1. SOP Neuroscore.

Supplemental Data 2. Composite phenotype scoring system for cerebellar ataxia.

Supplemental Data 3. Results statistical analyses. Overview of results from the statistical analyses provided per figure.

**Figure S1.**
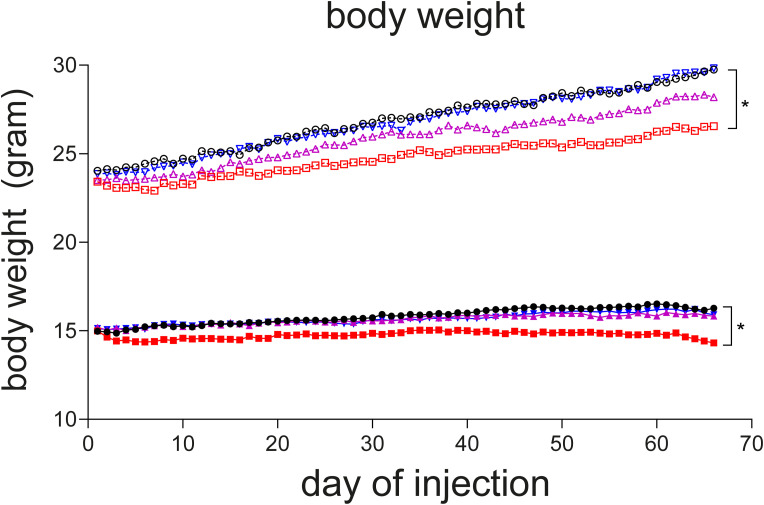
TUDCA decreases body weight gain in WT and VWM mice, whereas 4-PBA and PDPD do not. WT (open symbols) and *2b4*^*he*^*2b5*^*ho*^ (VWM, closed symbols) mice were injected daily with placebo (black circles), 500 mg/kg TUDCA (red squares), 120 mg/kg 4-PBA (magenta triangles), or 6 mg/kg PDPD (blue inverted triangles) from an age of 7–8 wk onwards for 9–10 wk (n = 8 WT mice and n = 16 VWM mice per treatment). Body weight was assessed daily before the injection. Graph shows mean body weight of each group. The body weight over time of placebo-treated *2b4*^*he*^*2b5*^*ho*^ mice is significantly lower than in WT controls (*P* < 0.0001; repeated measures two-way ANOVA; not indicated). Statistical analyses examining treatment effects were analyzed per genotype with a repeated measures two-way ANOVA followed by Dunnett’s multiple comparison test, *P < 0.05.

**Figure 3.**
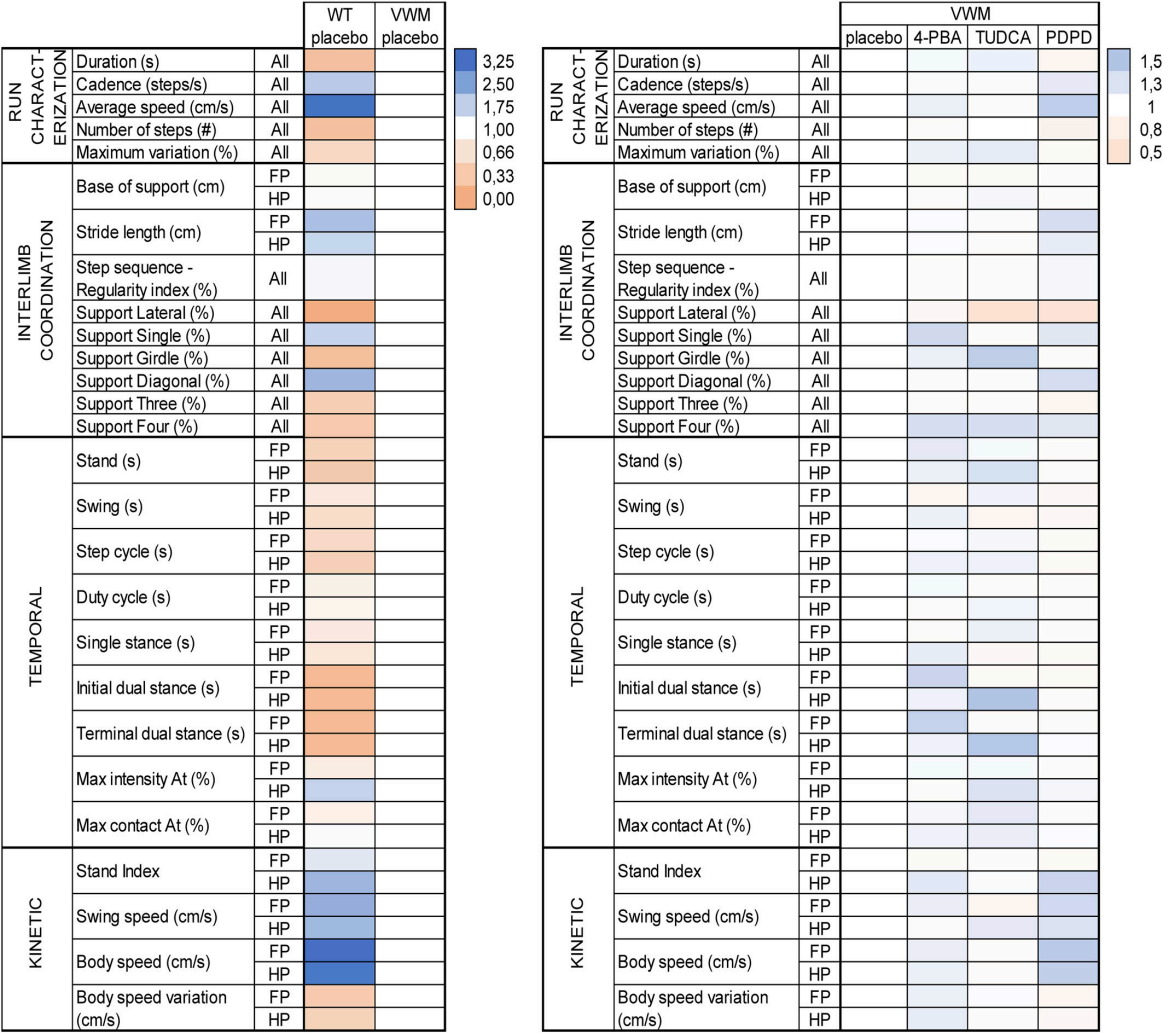
Heat maps showing genotype and treatment effects on CatWalk parameters. WT and *2b4*^*he*^*2b5*^*ho*^ (VWM) mice were injected daily with placebo, 500 mg/kg TUDCA, 120 mg/kg 4-PBA or 6 mg/kg PDPD from an age of 7–8 wk onwards for 9–10 wk (n = 8 WT mice and n = 16 VWM mice per treatment). CatWalk parameters are categorized in “run characterization,” “interlimb coordination,” “temporal,” and “kinetic.” Left hand heat map shows genotype effects in placebo-treated WT and VWM mice on CatWalk parameters. Right hand heat map shows treatment effects of 4-PBA, TUDCA, or PDPD in comparison with placebo-treated in VWM mice on CatWalk parameters. All data are normalized to placebo-treated VWM mice; all VWM placebo animals are ranked as 1. Range of the two scale bars differs. Color schemes are based on minimal score, 50th percentile (median), and maximal score of either heat map. How WT and VWM animals perform in relation to each other is shown in a separate heat map (Fig S2).

**Figure S2.**
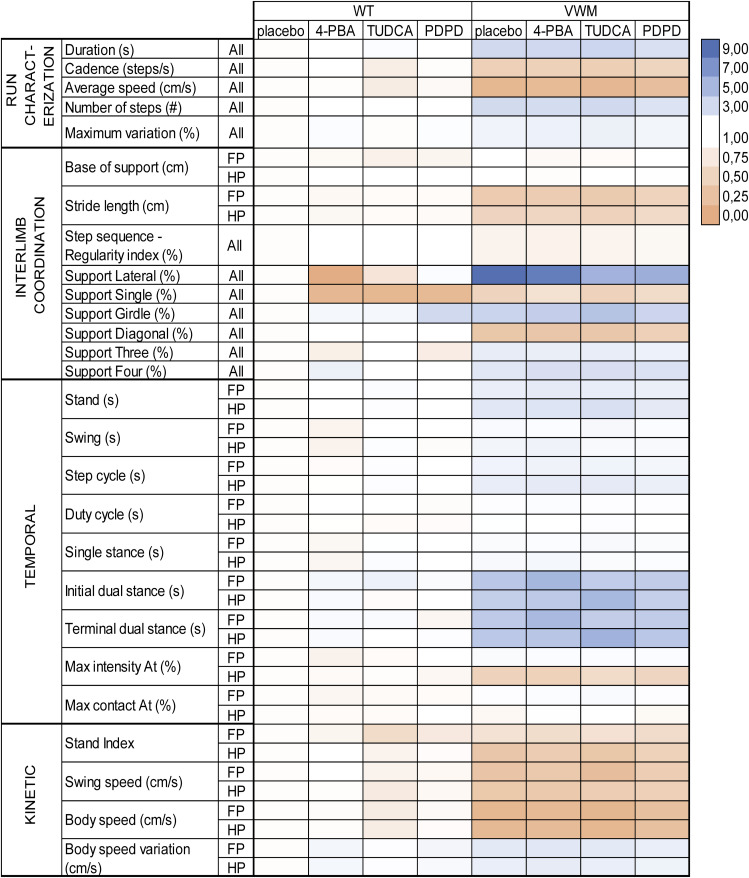
Heat map showing genotype and treatment effects on CatWalk parameters. WT and *2b4*^*he*^*2b5*^*ho*^ (VWM) mice were injected daily with placebo, 500 mg/kg TUDCA, 120 mg/kg 4-PBA or 6 mg/kg PDPD from an age of 7–8 wk onwards for 9–10 wk. Each treatment group consisted of n = 8 WT and n = 16 VWM mice. Heat map shows genotype and treatment effects of placebo, 4-PBA, TUDCA, or PDPD in WT and VWM mice on CatWalk parameters using the same scale bar. All data are normalized to WT placebo; all WT placebo animals are ranked as 1. CatWalk parameters are categorized in “run characterization,” “interlimb coordination,” “temporal,” and “kinetic.” Color scheme is based on minimal score, 50th percentile (median), and maximal score.

Body weight was reduced in VWM mice compared with WT mice. Daily injections with TUDCA reduced average body weight gain in WT mice by 12% (*P* = 0.0211) and in VWM mice by 11.3% (*P* = 0.0462) compared with placebo (Fig S1A). 4-PBA and PDPD did not affect body weight in WT or VWM mice. PDPD improved balance beam performance of VWM mice with 46% (*P* = 0.0153; Fig 2C), while TUDCA and 4-PBA did not. In VWM mice, 4-PBA and TUDCA did not statistically significantly improve CatWalk performance, but some temporal parameters worsened slightly (Fig 3). PDPD slightly ameliorated motor behavior on 15 out of 42 parameters; statistical significance was not reached (Figs 3 and S2). TUDCA altered four out of 60 gait parameters of the CatWalk tests in VWM mice, two of which improved and two of which worsened motor behavior. 4-PBA and TUDCA altered several gait parameters in WT mice, but without a consistent direction of effects (Supplemental Data 3).

### 4-PBA, TUDCA, or PDPD do not ameliorate neuropathological hallmarks of VWM

**Figure S3.**
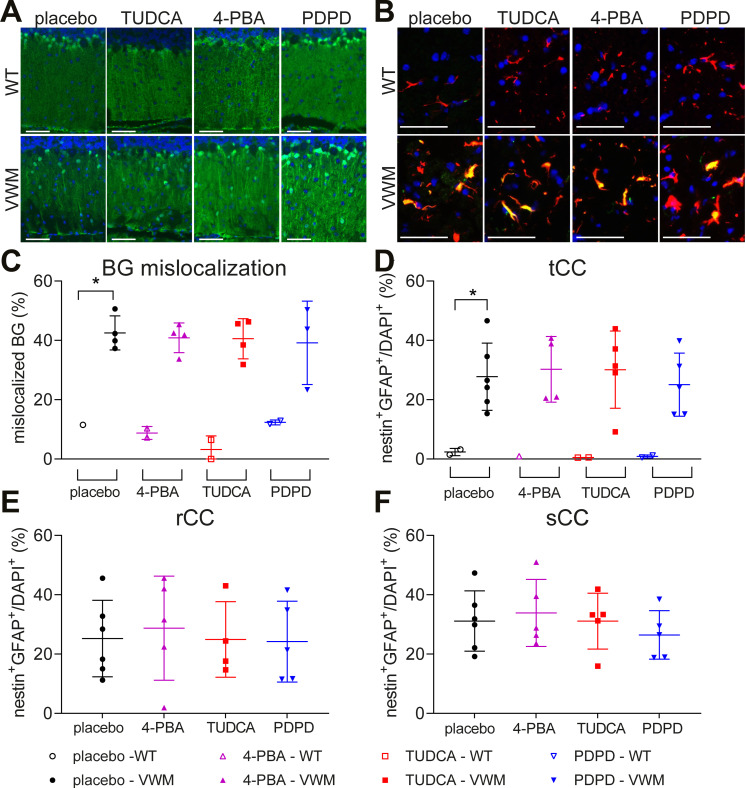
Bergmann glia mislocalization and the percentage of immature astrocytes are not ameliorated by 4-PBA, TUDCA, or PDPD. WT (open symbols, n = 1–2 per treatment) and *2b4*^*he*^*2b5*^*ho*^ (VWM, closed symbols, n = 3–6 per treatment) mice were injected daily with placebo (black circles), 500 mg/kg TUDCA (red squares), 120 mg/kg 4-PBA (magenta triangles), or 6 mg/kg PDPD (blue inverted triangles) from an age of 7–8 wk onwards for 9–10 wk. **(A, C)** Sagittally cut brain sections were subjected to immunostaining for S100β (green) and nuclear staining with DAPI (blue) (A) to determine the number of Bergmann glia (BG, double positive for S100β, and DAPI) positioned in the molecular and Purkinje layers (C). **(B, D, E, F)** In addition, sagittally cut brain sections were stained for nestin (green), GFAP (red), and nuclei (DAPI, blue) to count of nestin–GFAP double-positive astrocytes (orange) and DAPI-positive nuclei (B) in the total corpus callosum (tCC, (D)), divided in rostrum (rCC, (E)) and splenium (sCC, (F)). Graph (C) shows individual and mean percentages of mislocalized Bergmann glia ± SD. The percentage of mislocalized Bergmann glia in placebo-treated *2b4*^*he*^*2b5*^*ho*^ mice is significantly higher than in WT controls (*P* < 0.001; *t* test). Treatment effects on Bergmann glia mislocalization were assessed per genotype with a nested one-way ANOVA followed by Dunnett’s correction. Graphs (D, E, F) show individual and mean percentages ± SD of nestin–GFAP double-positive astrocytes in the tCC per genotype and treatment (D) or in rCC (E) or sCC (F) in VWM mice per treatment. Statistical analysis examining WT–VWM differences in placebo-treated mice for the tCC were performed with an unpaired *t* test (*P* = 0.024). Treatment effects on nestin–GFAP double-positive astrocytes were assessed with a one-way ANOVA followed by post hoc Dunnett’s correction per region of the corpus callosum. White bars, 50 μm.

**Figure S4.**
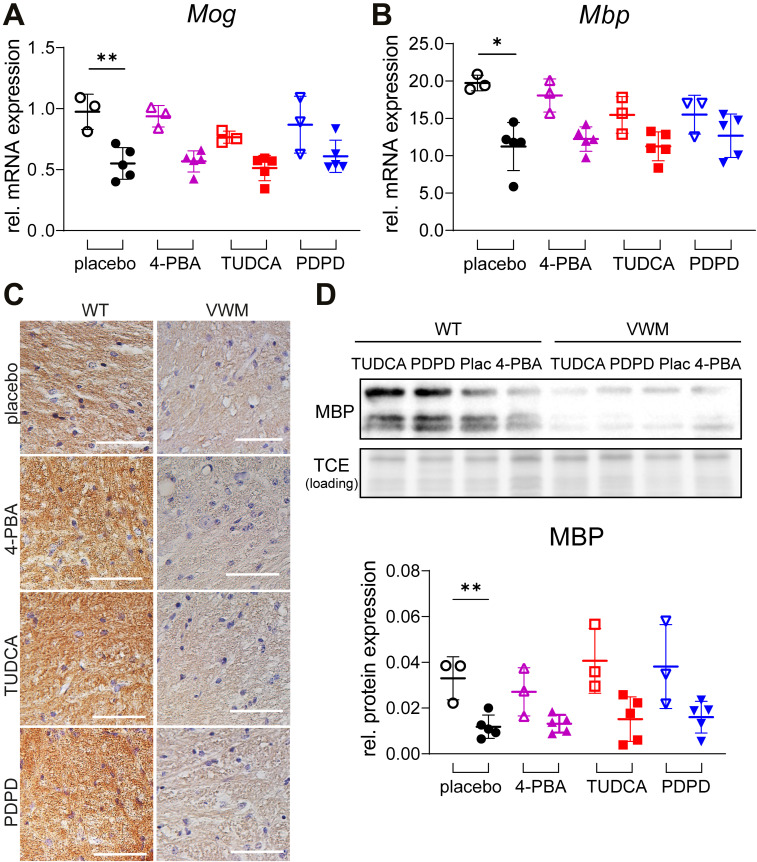
None of the compounds ameliorate myelin pathology in the cerebellum of VWM mice. WT and *2b4*^*he*^*2b5*^*ho*^ (VWM) mice were injected daily with placebo (black circles), 500 mg/kg TUDCA (red squares), 120 mg/kg 4-PBA (magenta triangles), or 6 mg/kg PDPD (blue inverted triangles) from an age of 7–8 wk onwards for 9–10 wk. **(A, B)** *Mog* and *Mbp* mRNA expression in cerebella from n = 3 WT and n = 5 *2b4*^*he*^*2b5*^*ho*^ VWM per treatment group were quantified with qRT-PCR (*Hprt* as reference). **(C)** Sagittally cut brain sections of n = 2 WT and n = 3 VWM mice per treatment were stained for MOG (brown) and counterstained with H&E (purple, indicating nuclei). Images show representative staining’s per indicated treatment. Images represent one mouse per condition. White bars, 50 μm. **(D)** Western blot for MBP was performed on n = 3 WT and n = 5 VWM cerebella per treatment group, respectively. Three MBP isoforms can be detected. Graph depicts relative protein expression of MBP. Graphs indicate individual data points and means ± SD. **(A, B, D)** Statistical analyses examining WT-VWM differences in placebo-treated mice were performed with an unpaired *t* test for *Mog* (A) and MBP (D), and with a Mann–Whitney test for *Mbp* (B). In comparison with WT mice, the levels of *Mog* (*P* = 0.0026; not indicated), *Mbp* (*P* = 0.0179; not indicated), and MBP (*P* = 0.0027; not indicated) were significantly decreased in VWM mice. **(A, B, D)** Treatments effects were statistically analyzed per genotype with a one-way ANOVA followed by post hoc Dunnett’s correction for *Mog* (A), *Mbp* (B), and MBP (D). *Mog* and *Mbp* mRNA levels increased subtly and consistently in PDPD-treated VWM mice relative to the placebo group (+10% and +13%).

**Figure S5.**
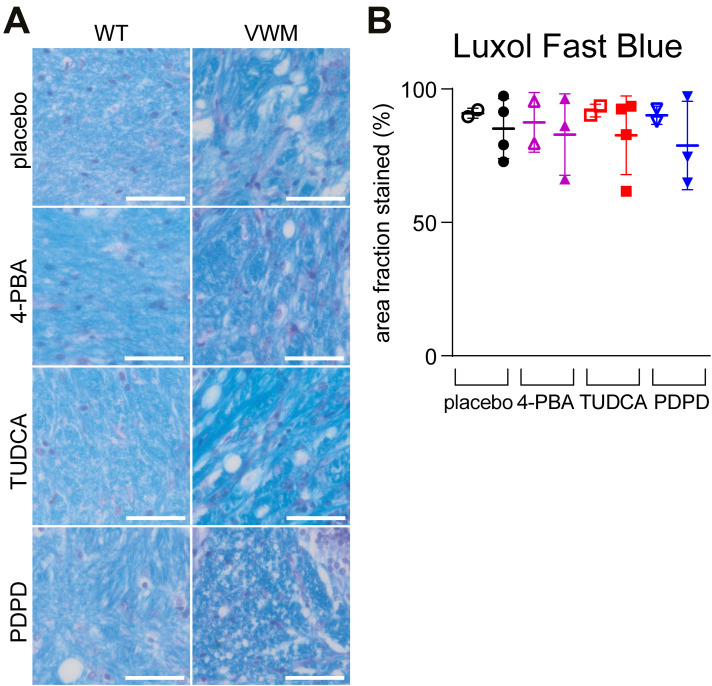
None of the compounds ameliorate cerebellar white matter vacuolization in VWM mice. WT and *2b4*^*he*^*2b5*^*ho*^ (VWM) mice were injected daily with placebo (black circles), 500 mg/kg TUDCA (red squares), 120 mg/kg 4-PBA (magenta triangles), or 6 mg/kg PDPD (blue inverted triangles) from an age of 7–8 wk onwards for 9–10 wk. **(A)** Sagittally cut brain sections of n = 2 WT and n = 4 VWM mice per treatment were stained for LFB (blue, indicating myelin) and counterstained with periodic acid-Schiff (purple, indicating nuclei). Images show representative stainings per indicated treatment. Images represent one mouse per condition. White bars, 50 μm. **(B)** Graph depicts average area fraction (%) of LFB positive pixels in cerebellar white matter per mice. Graph indicates individual data points and means ± SD. Genotype and treatment effects on the area fraction of LFB positive pixels were statistically assessed with a nested one-way ANOVA followed by post hoc Sidak’s correction (*P* = 0.92).

Neuropathological hallmarks in VWM mice include mislocalized Bergmann glia in the molecular layer of the cerebellum, increased numbers of nestin–GFAP double-positive astrocytes in the corpus callosum, and reduced expression of mature oligodendrocyte markers in myelin (Dooves et al, 2016, 2018; Abbink et al, 2019). These hallmarks were evident in placebo-treated VWM mice and not in placebo-treated WT mice (Figs S3A and B and S4). Daily injections with 4-PBA, TUDCA, or PDPD did not restore Bergmann glia localization (*P* = 0.9685; Fig S3A and C) or cause a statistically significant reduction of the number of nestin–GFAP double-positive immature astrocytes in the total corpus callosum (*P* = 0.8851; Fig S3B and D), rostral CC (*P* = 0.9600; Fig S3E), or splenial CC (*P* = 0.6939; Fig S3F) in VWM mice. The mean number of nestin–GFAP double-positive astrocytes in the splenium of VWM mice was decreased by 15% in PDPD-treated VWM mice in comparison with the placebo group, which was not observed for TUDCA or 4-PBA; statistical significance was not reached. The treatments did not alter myelin pathology in VWM mice (Figs S4 and S5). mRNA levels of the mature oligodendrocyte markers *Mog* and *Mbp* were significantly decreased in the cerebella of placebo-treated VWM mice compared with WT mice (*P* = 0.0026 and *P* = 0.0179, respectively). These levels did not statistically differ among VWM mice injected with placebo, 4-PBA, TUDCA, or PDPD (*P* = 0.6219, Fig S4A and *P* = 0.7468, Fig S4B). Immunohistochemistry for mature oligodendrocyte marker MOG showed that MOG levels in VWM mice were decreased compared with WT mice and were not improved by 4-PBA, TUDCA, or PDPD injections (Fig S4C). MBP protein levels were not statistically significantly different in VWM animals treated with placebo or the ER-targeting compounds (Fig S4D). The biggest effect on MBP levels was observed in PDPD-treated VWM animals (*P* = 0.6507, +35%). White matter vacuolization in cerebella of VWM mice was assessed with luxol fast blue (LFB) histochemistry (Fig S5A). The area fraction of LFB positive pixels varied considerably per mouse and statistically significant genotype or treatment effects were not observed (Fig S5B). In general, these neuropathological experiments did not reveal effects in WT brain by any of the tested compounds (Figs S3, S4, and S5).

### 4-PBA, TUDCA, or PDPD do not significantly affect the ISR in VWM mice

**Figure 4.**
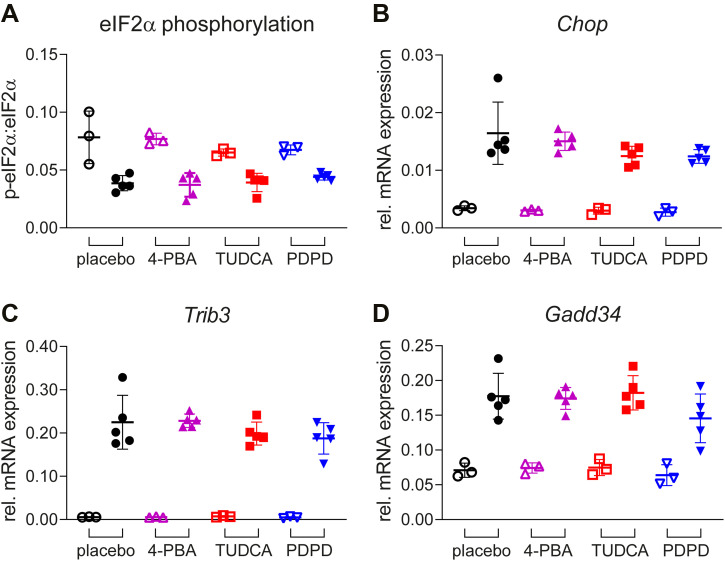
None of the compounds affect ISR deregulation in VWM mouse cerebella. WT (open symbols) and *2b4*^*he*^*2b5*^*ho*^ (VWM, closed symbols) mice were injected daily with placebo (black circles), 500 mg/kg TUDCA (red squares), 120 mg/kg 4-PBA (magenta triangles) or 6 mg/kg PDPD (blue inverted triangles) from an age of 7–8 wk onwards for 9–10 wk. **(A, B, C, D)** eIF2α phosphorylation (A) and ISR mRNA expression (*Chop*, *Trib3*, and *Gadd34*; (B, C, D)) in cerebella from n = 3 WT and n = 5 *2b4*^*he*^*2b5*^*ho*^ VWM per treatment group were quantified with Western blot and qRT-PCR (*Hprt* as reference), respectively. Protein and RNA samples were derived from the same cerebellar lysate. Graphs indicate individual data points and means ± SD. Shown ISR markers differ significantly in placebo-treated WT versus placebo-treated VWM mice (*P* < 0.05; not indicated). Statistical analyses examining WT–VWM differences in placebo-treated mice were performed with an unpaired *t* test with Welch’s correction for eIF2α phosphorylation and *Trib3*, Mann–Whitney test for *Chop*, and an unpaired *t* test for *Gadd34*. Treatments effects were analyzed per genotype with a one-way ANOVA followed by post hoc Dunnett’s correction, except for *Chop* in VWM mice which was analyzed with a Kruskal–Wallis test showing a general effect of treatment (*P* = 0.0358) followed by a post hoc Dunn’s correction yielding no significant differences for any compound.

Levels of phosphorylated eIF2α are low in VWM mouse brain relative to WT mouse brain (Abbink et al, 2019; Witkamp et al, 2022). Neither of the tested compounds altered the levels of phosphorylated eIF2α in cerebella of VWM or WT animals as compared with placebo controls (Fig 4A). The tested compounds also did not alter expression levels of ISR mRNAs in WT or VWM cerebella as compared with placebo treatment (Fig 4B–D). *Chop* mRNA levels in VWM animals were affected by treatment (*P* = 0.0358) with 4-PBA causing a small upregulation (+6%), and TUDCA (−50%) and PDPD (−54%) causing a down-regulation, although post hoc testing yielded no significant results. Despite these small effects on *Chop*, the expression of CHOP-regulated mRNAs *Trib3* and *Gadd34* did not statistically differ amongst VWM mice, irrespective of treatment (Fig 4). When focusing on the effects of PDPD in VWM mice, subtle and consistent reductions were observed in mean *Trib3* and *Gadd34* mRNA relative to the placebo group. TUDCA altered these levels also subtly, but not consistently in the same direction.

### Target engagement

**Figure S6.**
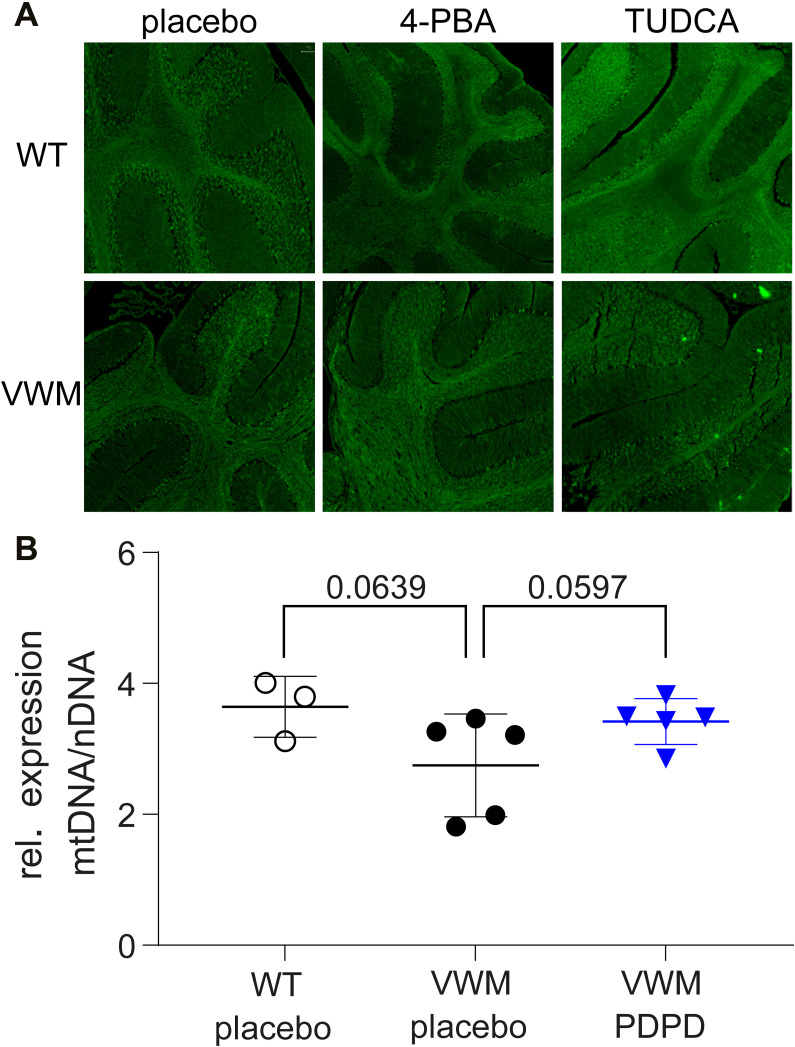
Target engagement of the compounds in VWM mice cerebella. WT and *2b4*^*he*^*2b5*^*ho*^ (VWM) mice were injected daily with placebo, 500 mg/kg TUDCA, 120 mg/kg 4-PBA, or 6 mg/kg PDPD from an age of 7–8 wk onwards for 9–10 wk. **(A)** Sagittally cut brain sections were stained with thioflavin T. **(B)** Mitochondrial DNA levels were quantified with qRT-PCR (nuclear DNA as reference) in cerebella from n = 3 placebo-treated WT (open black circles) and n = 5 placebo-treated VWM (closed black circles) and n = 5 PDPD-treated VWM (closed blue inverted triangles) mice. Data were analyzed with unpaired *t* tests. Graphs indicate individual data points and means ± SD.

The lack of a convincing ameliorating effect of any of the compounds on the VWM mice could indicate that the compounds failed to fully modulate their intended targets at the administered dose levels. To assess target engagement of 4-PBA and TUDCA, histological staining of brain sections with thioflavin T was performed to detect misfolded proteins as indicator of ER stress (Beriault & Werstuck, 2013). Increased levels of misfolded proteins were not found in VWM mice as compared with WT mice and levels were not affected by treatment (Fig S6A). To assess target engagement of PDPD, mitochondrial DNA (mtDNA) levels were quantified in cerebella of placebo- and PDPD-treated mice. MtDNA levels in placebo-treated VWM mouse cerebella were lower than in placebo-treated WT (*P* = 0.0639, −25%). PDPD injections normalized the mtDNA levels in VWM mice (*P* = 0.0597, +24%), as compared with placebo injections (Fig S6B).

### The CAS represents disease course of VWM mice better than the neuroscore

In the present study, two scoring methods for neurological decline were compared with determine which protocol assessed the VWM disease course most accurately in mice. The neuroscore detected the first signs of decline in VWM mice in week 6 of the experiment and the CAS in week 3 (Fig 1A–C). The gait performance of VWM mice was affected first, followed by their pelvic tilt (Fig 1D), ledge performance, and hind limb clasping (Fig 1C). Balance beam performance correlated better with the CAS of the last day (*r* = 0.9049, *P* < 0.0001) than with the neuroscore of the last day (*r* = 0.9000, *P* < 0.0833) of the experiment (data not shown). Thus, the CAS represents the phenotypic disease course of VWM mice better than the neuroscore.

## Discussion

Previous studies showed that improving ISR regulation with compounds that increase eIF2B activity are promising for VWM treatments (Abbink et al, 2019; Wong et al, 2019). The present study assessed the effects of three additional compounds on the disease progression in VWM mice.

PDPD, targeting a part of the ISR downstream of eIF2B, showed subtle amelioration of ataxia in VWM mice when assessed with the CAS protocol and the balance beam. This effect was not observed with the neuroscore protocol. Furthermore, a small, but consistent ameliorating effect by PDPD was observed in several CatWalk parameters displayed by VWM mice, supporting the positive findings in the CAS and balance beam tests. Probably, reaching statistical significance was hampered because of the multitude of Catwalk parameters, dictating a large number of comparisons. Multiple testing correction might in this case result in smaller effects being dismissed in statistical analyses. Amelioration of neuropathological hallmarks or the deregulated ISR in VWM mouse brain was not observed for PDPD, although the mean number of immature astrocytes in the splenium and the mean levels of ISR mRNAs in cerebella were subtly reduced VWM mice. Interestingly, PDPD normalized the mtDNA level in VWM mouse cerebella. It has been reported that PDPD has protective effects on mitochondrial dysfunction (Naia et al, 2021). Here, we show ameliorating effects of PDPD on the mtDNA levels, but also show that the clinical phenotype in PDPD-treated animals only marginally improved. Possibly, the mitochondrial deficit in VWM is not fully recapitulated by mtDNA levels, or it does not contribute much to neurological decline. Still, target engagement by PDPD was detected in VWM mice, although the statistical analysis only showed a trend, likely because of the small sample size. Given PDPD’s very short half-life in mice, increasing the dose frequency from once a day till several times a day could prolong its effect and improve results in VWM mice (Francardo et al, 2019). Still, in a Huntington disease mouse model, in which the ISR is activated, neuroprotective effects were already found with a dosage of 5 mg/kg (Squitieri et al, 2015). Also, higher doses can activate the dopamine D2 receptor (Dyhring et al, 2010), potentially leading to undesirable off-target effects (Missale et al, 1998; Borovac, 2016). In conclusion, PDPD has a slight beneficial effect on the neurological phenotype in VWM mice, suggesting that S1R effects do not greatly contribute to clinical signs. The deregulated expression of ATF4 and CHOP in VWM brain results in more than the S1R effects alone (Han et al, 2013; Atzmon et al, 2018; Abbink et al, 2019). These additional ATF4- and CHOP-regulated effects are not ameliorated by PDPD treatment. Our results indicate that isolated targeting of the S1R effects only subtly ameliorates VWM clinical and pathological hallmarks in VWM mice. PDPD’s half-life in adult human healthy volunteers is longer than in mice and ranges between 6 and 15 h, depending on the genetic polymorphism in CYP2D6 (Lindskov Krog et al, 2013). After adequate translational pharmacokinetic-pharmacodynamic modeling, the potential small beneficial effect of PDPD observed in VWM mice might be investigated in combination treatment for VWM patients. For example, PDPD might be combined with a compound that has significant side effects, so that the dosage of the latter drug can be decreased to reduce the unwanted effects. It can also be used in combination with another drug with incomplete efficacy in VWM to enhance each other’s treatment effects.

4-PBA and TUDCA, the two compounds that act upstream of eIF2B, did not have any ameliorating effects on the motor skills, neuropathological hallmarks, or deregulated ISR of VWM mice. With the given dosages, we even noticed slightly worsening effects on Catwalk gait parameters in VWM mice and adverse effects in both genotypes (body weight and acute temporary reaction to TUDCA). Analyses of target engagement by 4-PBA or TUDCA in WT and VWM mouse brains showed similar levels of misfolded proteins, irrespective of genotype or treatment, confirming the absence of increased ER stress in VWM mouse brain (Abbink et al, 2019). Target engagement confirmation of TUDCA and 4-PBA was not possible. Still, we need to consider that for each compound the optimal dosage for ISR targeting was determined on the basis of available preclinical literature (Kaemmerer et al, 2001; Keene et al, 2002; Qi et al, 2004; Zhang et al, 2016; Bhardwaj et al, 2019). A dose level was selected that showed efficacy for an intracranial target and acceptable toxicity. These findings suggest that targeting the ISR upstream of eIF2B is not effective for clinical amelioration in VWM. A previous study on Sephin1 treatment in VWM mice came to the same conclusion by showing that Sephin1 reduces brain eIF2α phosphorylation levels without any ameliorating effects on the clinical phenotype (Witkamp et al, 2022). It is important to realize, however, that the VWM mice only recapitulate the chronic disease course and not the acute neurological deteriorations observed in patients (Hamilton et al, 2018; Wisse et al, 2022). In VWM patients, the stress conditions provoking acute neurological decline are known eIF2α-phosphorylating events. Currently, preventive measures are taken in patients to avoid those stress conditions. Perhaps, a maintenance dose of 4-PBA or TUDCA will help prevent acute episodes of neurological decline following stress. Alternatively, these compounds may be applied as a potential, acute treatment in the case of such episodes.

Two different scoring methods for neurological decline in VWM mice were compared in this study: the neuroscore (Hatzipetros et al, 2015), which was the standard method in our previous studies, and the CAS (Guyenet et al, 2010). The CAS protocol showed an earlier onset of ataxia in VWM mice than the neuroscore. In addition, severity of the neurological decline was more accurately determined with CAS, probably because this test is based on four parallel scores instead of one sequential score, which is the case for the neuroscore. Only the CAS significantly correlated with motor function, as assessed by balance beam performance. We recommend that future preclinical trials replace the neuroscore by the CAS as a measure for neurological decline, as it is a more refined phenotypic scoring method for VWM mice.

In conclusion, treatments aimed at eIF2B or downstream ISR components may be more advantageous for improving chronic neurological deterioration in VWM patients than those aimed at the ER or other targets upstream of eIF2B. If compounds targeting the ISR upstream of eIF2B can prevent or ameliorate acute decline should be assessed in appropriate VWM models.

## Materials and Methods

### Animals

Experiments were performed with *2b4*^*he*^*2b5*^*h*o^ (VWM) mice, which are heterozygous for eIF2Bδ Arg484Trp and homozygous for eIF2Bε Arg191His (Dooves et al, 2016; Abbink et al, 2019). WT C57BL/6J mice were included as healthy controls. All mice were bred and housed as specific pathogen free animals in individually ventilated cages with standard nesting material and gnawing sticks. Mice were weaned at P28 and kept at a 12-h light/dark cycle with food and water provided ad libitum. Animal experiments were performed in compliance with the Dutch and European law and with approval of the local animal care and use committee of the VU University (license CCD AVD1120020172804, work-protocol 2804-NEU19-14A3). All methods have been reported in accordance with recommendations in the ARRIVE guidelines. There were seven breeding cycles in total. Per breeding cycle, WT and VWM mice were evenly assigned to all treatment groups based on their initial body weight to prevent a body weight bias. During the experiment, mice were solitarily housed.

### Compound preparations

TUDCA (Axenic) was dissolved in PBS to 50 mg/ml. PDPD (Prilenia) was dissolved in water-for-injection (WFI) to 0.6 mg/ml. 4-PBA (Sigma-Aldrich) was dissolved in sodium hydroxide and diluted with saline to 12 mg/ml. The vehicles PBS or WFI were used as placebo. The dosage for each compound that is optimal for ISR targeting was determined on the basis of available preclinical literature (Kaemmerer et al, 2001; Keene et al, 2002; Qi et al, 2004; Squitieri et al, 2015; Zhang et al, 2016; Sahlholm et al, 2018; Bhardwaj et al, 2019; Francardo et al, 2019). A dose level was selected for each compound that showed efficacy for an intracranial target and acceptable toxicity. For each compound, the brain tissue-to-plasma partition coefficients (KP) were above 1, and preferably ISR effects (decrease in phosphorylated eIF2α, CHOP, ATF4, or other factors) were also observed, suggesting that the drugs accumulate in brain tissue and show efficacy at 500 mg/kg TUDCA, 6 mg/kg PDPD, or 120 mg/kg 4-PBA dosing.

### Long-term treatment with TUDCA, 4-PBA, or PDPD and clinical assessments

The study design follows that of previous studies (Abbink et al, 2019; Witkamp et al, 2022). After an acclimatization period of ∼7 d, male WT and VWM mice were injected daily into the intraperitoneal cavity with placebo, 500 mg/kg TUDCA, 6 mg/kg PDPD, or 120 mg/kg 4-PBA from an age of 7–8 wk. Each treatment group consisted of 8 WT and 16 VWM mice, n = 96 mice in total. Injections were placed alternating on left- or right-hand side of the abdominal midline. Half of the placebo group was injected with PBS and half with WFI. Injections with TUDCA caused temporary discomfort in WT and VWM mice, as assessed with the mouse grimace scale (Langford et al, 2010). Discomfort lasted up to a maximum of 90 min after each injection. Body weight was monitored daily in all mice. Neurological deterioration was scored weekly with two different methods that determine a neuroscore (Hatzipetros et al, 2015) or a CAS. Protocols for neuroscore and CAS are provided (Supplemental Datas 1 and 2). The CAS is based on the composite phenotype scoring system from Guyenet et al (2010) and consists of four different measures recorded on a scale of 0–3 with a combined total of 0–12 for all four measures (Guyenet et al, 2010). We adapted this method for the disease phenotype in VWM mice by exchanging the kyphosis parameter for a pelvic tilt parameter (Fig 1D). We also adjusted the scale for gait to better represent our VWM mouse model by adding the 1.5 score (tremor and hammer toes). Hence, the CAS consists of a combination of scores for the degree of hind limb clasping, gait, pelvic tilt, and ledge test. Motor skills were assessed on a 1.2-cm-wide balance beam after training on a 2.6-cm-wide beam (Dooves et al, 2016), and on the CatWalk XT 10.6 (Abbink et al, 2019) after 9–10 wk of injections. Mice were terminated by cervical dislocation or PFA perfusion after the CatWalk. Tissues were collected for postmortem analyses. CatWalk data were included only if mice had a minimum of six consecutive steps without pauses or turns. Data were analyzed by researchers blinded to genotype and treatment.

### Postmortem analyses

Immunostainings to detect MOG, S100β or GFAP, and nestin were performed as described (Witkamp et al, 2022). Counts were performed by researchers blinded to genotype and treatment. Thioflavin T (ab120751; Abcam) staining performed on deparaffinized mouse brain sections were performed as described for thioflavin S (den Haan et al, 2018). LFB staining was performed on deparaffinized mouse brain sections using in-house standard operating protocol. RNA and protein quantifications in cerebella were performed with qRT-PCR and Western blot as described (Witkamp et al, 2022), using anti-MBP antibody MAB387 (1:100; Millipore). mtDNA in Trizol-extracted cerebella was quantified with oligonucleotides that amplify *mtDNA* genes encoding 12sRNA (Atzmon et al, 2018), 16sRNA (Quiros et al, 2017), ND1 (Quiros et al, 2017), and CO1 (Guo et al, 2009). Nuclear DNA was quantified as reference, using the oligonucleotides for two nuclear encoded genes: *HK2* (Quiros et al, 2017) and *NDUFV1* (Guo et al, 2009). The amount of input template in the qRT-PCR differed between the reactions that detected mtDNA (2.5 ng per reaction) and Nuclear DNA (250 ng per reaction) to stay within the linear range of detection. The qRT-PCR was performed as described (Quiros et al, 2017).

### Statistical analyses

All animals were included for statistical analyses. Results from qRT-PCR and Western blot were corrected for session variation with the software program Factor (Ruijter et al, 2006). Variation in treatments or conditions is not corrected in this program. ImageJ2 was used to quantify the area fraction of LFB positive pixels in cerebellar white matter in three to five pictures per mice. Statistical analyses were performed with GraphPad Prism 9.3.1 software. The results from each statistical analysis are listed per figure in Supplemental Data 3. Differences were considered significant when *P* < 0.05. There were no statistical differences found in any parameter tested between WFI or PBS placebo-treated mice within the same genotype, as examined with an unpaired *t* test or an appropriate nonparametric alterative. Therefore, WFI- and PBS-treated mice were treated as one placebo group per genotype. For each experiment, placebo-treated WT and VWM animals were compared with identify statistically significant differences in VWM disease parameters. Next, treatment effects of PDPD, TUDCA, and 4-PBA were examined for these disease parameters in VWM and, if applicable, WT animals separately with a one-way ANOVA, two-way ANOVA, *t* test or appropriate nonparametric alternative, as indicated in figure legends. CatWalk performance of all treatment groups was analyzed using the software program R, as previously described (Supplemental Data 3) (Witkamp et al, 2022). Individual CatWalk parameters were categorized as described (Caballero-Garrido et al, 2017).

## Data Availability

The datasets generated during and/or analyzed during the present study are available from the corresponding author on reasonable request or are included in this published article (Supplemental Datas 3 and 4).

Supplemental Data 4. Raw data CatWalk. Overview of mean values per individual mouse per CatWalk parameter.

## Supplementary Material

Reviewer comments
