# Supplementary material for: Pridopidine subtly ameliorates motor skills in a mouse model for vanishing white matter
Source: Life Sci Alliance. 2024 Jan 3;7(3):e202302199. doi: 10.26508/lsa.202302199 (PMC10765115; doi:10.26508/lsa.202302199)
Supplement: Supplementary file 1 [file LSA-2023-02199_Supplemental_Data_1.docx]

**Data S1. SOP Neuroscore**

The neurological score employed a scale of 0 to 4 that was developed by observation at ALSTDI. Criteria used to assign each score level were:

Score Criteria

**0,0)** Full extension of hind legs away from lateral midline when mouse is suspended by its tail, and mouse can hold this for 2 seconds, suspended 2–3 times.

**0,5)** Partial extension of hind legs, not fully away from lateral midline.

**1,0)** Collapse of leg extension towards lateral midline (weakness)

**1,5)** Trembling of hind legs during tail suspension.

**2,0)** Toes curl under during walking of approx. 12 inches and/or trembling of hind legs.

**2,5)** Any part of foot is dragging along cage bottom/table*. **(Food provided in cage floor)**

**3,0)** Rigid paralysis or minimal joint movement, foot not being used for forward motion.

**3,5)** Mouse has trouble moving forward and keeping a upright position.

**4,0)** Mouse cannot right itself within 30 seconds from either side.

*If one hind leg is scored as 2, food pellets are left on bedding. If both hind legs are scored as 2, Nutra-Gel® (Bio-Serve #S4798) is provided as food in addition to food pellets on bedding and a long sipper tube is placed on the water bottle.
