# Supplementary material for: Pridopidine subtly ameliorates motor skills in a mouse model for vanishing white matter
Source: Life Sci Alliance. 2024 Jan 3;7(3):e202302199. doi: 10.26508/lsa.202302199 (PMC10765115; doi:10.26508/lsa.202302199)
Supplement: Supplementary file 2 [file LSA-2023-02199_Supplemental_Data_2.docx]

**Data S2. Composite phenotype scoring system for cerebellar ataxia**

**Aim**

Scoring of cerebellar ataxia phenotype by combining the scores of 4 individual tests.

**Materials**

• IVC cage for ledge test

**Health and safety**

Does not apply

**Method**

The final score is determined by combining the scores of the 4 individual tests (hindlimb clasping, gait, pelvic tilt & ledge test). The individual tests are scored on a scale of 0-3. For the ledge test, the first week is of testing is seen as the ‘habituation phase’.

Hindlimb clasping test

• Grasp the tail near its base and lift the mouse clear of all surrounding objects.

• Observe the hindlimb position for 10 seconds (see figure 1) and give it a score:

0= the hindlimbs are consistently splayed outward, away from the abdomen

1= one hindlimb is retracted toward the abdomen for more than 50% of the time suspended

2= both hindlimbs are partially retracted toward the abdomen for more than 50% of the time suspended

3= its hindlimbs are entirely retracted and touching the abdomen for more than 50% of the time suspended

• Place the mouse back into its cage and record its hindlimb clasping score.

Gait test

• Remove the mouse from its cage and place it on a flat surface with its head facing away from the investigator.

• Observe the mouse from behind as it walks and give it a score:

0= the mouse moves normally, with its body weight supported on all limbs, with its abdomen not touching the ground, and with both hindlimbs participating evenly

1= it shows a tremor

1.5=it shows a tremor & walks high on its toes (hammertoes)

2= it shows a severe tremor, high on the toes, lowered pelvis, or the feet point away from the body during locomotion ("duck feet")

3= the mouse has difficulty moving forward and drags its abdomen along the ground

• Place the mouse back into its cage and record its gait score.

Pelvic tilt

• Place the mouse on a flat surface area.

• Observe the mouse as it walks and give it a score:

0= it can easily stretch/straighten its spine while walking & does not show a lowered pelvis, and the tail is held up

1= it has a mild pelvic tilt, but can stretch/straighten its spine and lift its tail up from the floor

2= it cannot stretch/straighten its spine & has persistent, but mild pelvic tilt

3= it shows clearly visible pelvic tilt while walking or sitting

Ledge test

• Lift the mouse from its home cage and place it on the ledge of the IVC test cage.

• IMPORTANT - During the habituation phase, which is in the first week, you should prevent that the mouse lowers itself in the case until it reached the other side of the ledge. If you see that the mouse wants to lower itself before that, lift the mouse by its tail and place it back on the ledge. Keep doing this until it reaches the other and of the ledge, then the mouse is allowed to lower itself.

• Observe the mouse as it walks along the cage ledge and lowers itself into the cage and give it a score:

0= it walks along the ledge without losing its balance, and lowers itself back into the cage gracefully, using its paws.

1= mouse loses its footing while walking along the ledge, but otherwise appears coordinated

2= mouse does not effectively use its hind legs, or lands on its head rather than its paws when descending into the cage

3= mouse (nearly) falls off the ledge while walking or attempting to lower itself, or shakes and refuses to move despite encouragement
